# Lymphoscintigraphy for Evaluation of Lymphedema Treatment: A Systematic Review

**DOI:** 10.7759/cureus.6363

**Published:** 2019-12-12

**Authors:** Antonio J Forte, Daniel Boczar, Maria T Huayllani, Xiaona Lu, Pedro Ciudad

**Affiliations:** 1 Plastic Surgery, Mayo Clinic Florida Robert D. and Patricia E. Kern Center for the Science of Health Care Delivery, Jacksonville, USA; 2 Plastic Surgery, Yale University, New Haven, USA; 3 Plastic, Reconstructive and Burn Surgery, Arzobispo Loayza National Hospital, Lima, PER

**Keywords:** lymphedema, lymphoscintigraphy, lymphedema surgery, microsurgery, lymphovenous bypass, biomedical imaging, treatment, image evaluation, nuclear medicine

## Abstract

Lymphoscintigraphy is a well-established radiologic examination to evaluate lymphatic function. We conducted a systematic review of the use of lymphoscintigraphy for evaluation of lymphedema treatment. We hypothesized that this radiologic examination could add relevant findings of treatment outcomes of lymphedema patients. We conducted a systematic review of articles in PubMed, without any time frame or language limitations, about the use of lymphoscintigraphy for the evaluation of lymphedema treatment. Articles were excluded if they investigated other uses of lymphoscintigraphy, such as diagnosis or prevention of lymphedema. Abstracts, presentations, reviews, and meta-analyses were also excluded. Of 101 potential articles found in the literature, 5 fulfilled our study eligibility criteria, and they were all case series. These articles included a total of 327 patients, most of whom had breast cancer-related lymphedema. Interventions included lymph node or vessel transfer (3 of 5 articles), complex decongestive therapy (1 of 5), and adipose-derived stem cell injection (1 of 5). The authors of these studies used lymphoscintigraphy to investigate the treatment functional outcomes, prognostic value, and complications. Lymphoscintigraphy detected lymphangiogenesis in transferred lymph nodes, and it was able to predict patient response to complex decongestive therapy. Studies that used lymphoscintigraphy to evaluate lymphedema treatment demonstrated its flexibility to provide various types of information. We hope this review will support future studies.

## Introduction and background

Lymphedema is a chronic condition that affects millions of people around the world, and it is characterized by tissue edema, inflammation, and fibrosis [1-3]. Incidence rates are high among people who undergo surgical treatment of solid tumors, and lymphedema affects approximately one in every six patients [4]. Although lymphedema is diagnosed clinically, lymphoscintigraphy is a well-established radiologic examination to evaluate and confirm the diagnosis, as well as to measure lymphatic function [5-6].

Still considered an incurable condition, lymphedema has challenged clinicians around the world to propose new treatment modalities [7]. In this scenario, imaging examinations that allow further assessment of lymphatic function, such as lymphoscintigraphy, could be useful to understand therapeutic responses [8]. Therefore, we conducted a systematic review of articles about the use of lymphoscintigraphy for the evaluation of patients undergoing lymphedema treatment. We hypothesized that this radiologic examination could add relevant findings of the treatment outcomes of lymphedema patients.

## Review

Methods

Search strategy: On October 30th, 2019, two reviewers (D.B. and M.T.H) independently searched the PubMed database, without any time frame or language limitations, for articles about the use of lymphoscintigraphy for evaluation of patients undergoing lymphedema treatment; initially, the title and abstract were screened, and then the full text was reviewed. Disagreements regarding article identification and final selection for inclusion in this study were resolved by another reviewer (A.J.F). The search was done with the following Medical Subject Heading (MeSH) terms: “breast cancer lymphedema” AND “lymphoscintigraphy.” The reference lists of the studies that fulfilled the study eligibility criteria were also examined, and we looked for articles not identified in our initial search. This current study followed the guidelines outlined in the Preferred Reporting Items for Systematic Reviews and Meta-Analyses (PRISMA).

Selection criteria: Studies eligible for inclusion reported data about the use of lymphoscintigraphy to evaluate lymphedema treatment. Therefore, we excluded articles that investigated other uses of lymphoscintigraphy, such as lymphedema diagnosis or prevention. Abstracts, presentations, reviews, and meta-analyses were also excluded.

Data extraction and processing: Extracted data included the year of publication, country, study design, level of evidence, population, intervention, use of lymphoscintigraphy, comparison measurements, and key findings regarding the evaluation of treatment outcomes with lymphoscintigraphy. Data extraction from articles, tables, and figures was performed by two reviewers (D.B. and M.T.H), and accuracy of data entry was confirmed by an additional reviewer (A.J.F).

Results

**Figure 1:**
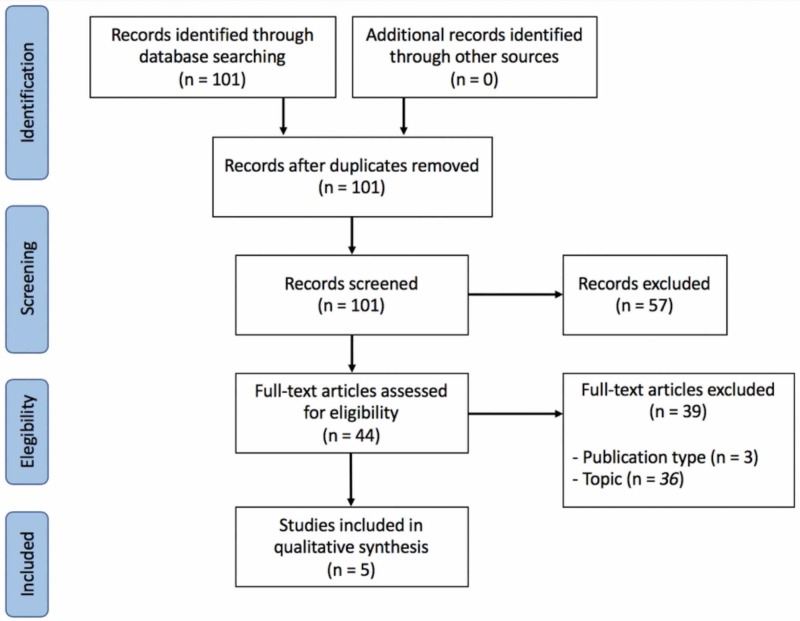
Preferred Reporting Items for Systematic Reviews and Meta-Analyses (PRISMA) Diagram

Study characteristics: Of 101 potential articles found in the literature, 5 fulfilled the study eligibility criteria (Figure 1 and Table 1). The use of lymphoscintigraphy to evaluate lymphedema treatment was described in only case series conducted in Asia (3 of 5 articles) and Europe (2 of 5). These articles included a total of 327 patients, most of whom had breast cancer-related lymphedema. Interventions included lymph node or vessel transfer (3 of 5 articles) [3,6,9], complex decongestive therapy (CDT) (1 of 5) [10], and adipose-derived stem cell (ADSC) injection (1 of 5) [7]. In most studies, lymphoscintigraphy was performed before and after treatment [3,6-7,9]. However, in one study, lymphoscintigraphy was performed only before treatment, with the aim of predicting clinical response to CDT [10]. The authors used the examination to investigate treatment functional outcomes, prognostic value, or complications.

**Table 1:** Summary of the Studies Abbreviations: BCRL, breast cancer related lymphedema; PLE, primary lymphedema; ALVT, autologous lymph vessel transplantation; VGLN, vascularized groin lymph node (VGLN); CDT, complex decongestive therapy; ADSCs, adipose derived stem cells; LCM, limb circumference measurement

Author	Year	Country	Study design	Level of evidence	Population		Intervention	Lymphoscintigraphy	Objective	Comparison	Key findings
Weiss et al. [3]	2015	Germany	Case series	Level II	177 patients	BCRL (169); PLE (2); Other cancers (6)	ALVT	Before and after intervention. Affected limb	Evaluate functional outcome	LCM	Agreement between lymphoscintigraphy and volume measurements.
Liu et al. [6]	2018	China	Case series	Level II	30 patients	BCRL	VGLN transfer	Before and after intervention. Affected limb	Evaluate functional outcome; Provide radiological evidence of lymphangiogenesis	LCM	Disagreement between lymphoscintigraphy and volume measurement.
Kim et al. [10]	2018	Korea	Case series	Level II	80 Patients	BCRL	CDT	Before intervention. Affected limb	Evaluate prognostic value	Electronic volume device	Lymphoscintigraphy predicted clinical response to CDT
Liu et al. [9]	2018	China	Case series	Level II	30 patients	BCRL	VGLN transfer	Post-intervention. Donor limb	Investigate complications	LCM	Agreement between lymphoscintigraphy and clinical findings. There was no donor limb lymphedema.
Toyserkani et al. [7]	2019	Denmark	Case series	Level II	10 patients	BCRL	ADSCs injection	Before and after intervention. Affected limb	Evaluate functional outcome	LCM	Agreement between lymphoscintigraphy and LCM. There was no significant change in lymphedema after intervention.

Evaluation of functional outcomes: Weiss et al. conducted a study to evaluate functional outcomes of autologous lymph vessel transplants in 177 lymphedema patients (172 women and 5 men; median age, 56 years) [3]. Patients were clinically and radiologically evaluated at four time points: before surgery; two weeks after surgery (T1); six to twelve months after surgery (T2); and 32 to 38 months after surgery (T3). At T1, T2, and T3, the mean percentage of volume reduction was correlated with the mean improvement of the transport index on lymphoscintigraphy by a factor of 2.64. Moreover, the radiologic evaluation showed persistent improvement in 19 patients who had more than eight years of follow-up [3].

Toyserkani et al. conducted the first human pilot study of the use of ADSCs in lymphedema treatment [7]. They recruited 10 patients (median age, 55 years) with breast cancer-related lymphedema who received injections of ADSCs into the axillary region associated with a scar-releasing, fat-grafting procedure. Patients were observed for one year, and quantitative lymphoscintigraphy was used to evaluate the functional outcome. Only minor transient complications (related to liposuction) were observed. Although they observed an improvement in patient-reported outcomes, clinical and radiological evaluations demonstrated the absence of improvement in the affected limbs [7].

Liu et al. conducted a study of 30 patients (mean age, 60 years) with breast cancer-related lymphedema who underwent vascularized groin lymph node transfer with the axilla as the recipient site [6]. At a mean (SD) follow-up of 22.11 (7.83) months, they noted a reduction in limb circumference in 70% of their cohort (*n*=21; mean [SD] reduction rate, 47.06% [27.92%]); however, this observation did not agree with the lymphoscintigraphy findings, which showed that only 37% of patients (*n*=11) had radiologic improvement. Interestingly, they pointed out radiologic evidence of lymphangiogenesis in at least four patients, whose transplanted lymph nodes appeared on the examination [6].

Evaluation of complications: Liu et al. conducted another analysis of the same 30 lymphedema patients who underwent vascularized groin lymph node transfer, as described above [9]. In their study, lymphoscintigraphy was used to investigate potential donor-site complications of lymph node harvest. They compared radiologic and clinical findings of donor limbs and nonoperated limbs. Patients were observed for a mean (SD) of 22.11 (7.83) months. Although some patients had seroma and transient thigh dysesthesia, none had clinically relevant lymphedema of the donor limb. In agreement with the clinical findings, lymphoscintigraphy demonstrated normal contrast uptake and absence of dermal backflow. The mean transport index was 3.32 for the donor limbs vs 2.04 for the nonoperated limbs [9].

Prognostic value:* *Kim et al. conducted a study of 80 patients (mean age, 51.2 years) with breast cancer-related lymphedema who were treated with CDT for one year [10]. Pretreatment lymphoscintigraphy findings were correlated with patient response to CDT to determine whether the examination has prognostic value. After one year of CDT, 50 patients were poor responders (<10% reduction in limb volume) and 30 were responders (>10% reduction in limb volume). Radiologic visualization of axillary lymph nodes and patient compliance were associated with greater response to CDT (odds ratio (95% CI), 21.33 (2.37-192.03)), compared with invisible axillary lymph nodes and poor compliance [10].

Discussion

In this systematic review, we noted that the scientific evidence about the use of lymphoscintigraphy to evaluate lymphedema treatment can be summarized in five case series, which included a total of 327 lymphedema patients. The examination was used in different ways, including to investigate functional outcomes, prognostic value, and identifying complications. Most studies pointed out that the radiologic findings of lymphoscintigraphy correlated well with those of the clinical evaluations [3,7,9]. The examination was valuable because it provided clinically useful information that otherwise would not have been obtained. Specifically, lymphoscintigraphy detected lymphangiogenesis in transferred lymph nodes, which reconnected with the lymphatic circulation [6], and it was able to predict patient response to CDT [10]. To our knowledge, this is the first systematic review to investigate the use of lymphoscintigraphy to evaluate lymphedema treatment.

We recognize that our study has multiple limitations common to systematic reviews, including the risk of bias in analyzing the data presented in each publication. Moreover, the search was conducted using only one database (PubMed) and was focused on the MeSH term “breast cancer lymphedema” (as described above in the Search Strategy section), which might explain why only clinical studies were found. We also did not include studies about the use of lymphoscintigraphy for harvesting lymph node flaps [11]. Despite that, we understand that our systematic review adds a relevant overview of the scientific evidence about the use of lymphoscintigraphy to evaluate lymphedema treatment.

## Conclusions

The studies that used lymphoscintigraphy to evaluate lymphedema treatment demonstrate its flexibility to provide various types of information. Study authors have used lymphoscintigraphy to evaluate functional outcomes, donor-site complications of lymph node harvest, and prognostic value of CDT. All studies (except 1) reported agreement between radiologic and clinical evaluations. We hope this review will support future studies to further delineate the clinical utility of lymphoscintigraphy for the evaluation of lymphedema treatment.
